# Supplementary material for: Transcriptomic Profiling of Meat Quality Traits of Skeletal Muscles of the Chinese Indigenous Huai Pig and Duroc Pig
Source: Genes (Basel). 2023 Jul 28;14(8):1548. doi: 10.3390/genes14081548 (PMC10454112; doi:10.3390/genes14081548)
Supplement: Supplementary file 1 [file genes-14-01548-s001.zip › Table S1. Primers used in qPCR for twenty-one genes..pdf]

Table S1. Primers used in qPCR for twenty-one genes.

Genes	Sequence（5'-to-3')
ANKRD1	*F:GGACCGAGAAGGAGATACCC*<br>*R:ATGCGAGAGGCTTTGTAGGA*
NR1D2	*F:CACGCTTGTGAAGGCTGTAA*<br>*R:ATACGACCAAACCGAACAGC*
MID1IP1	*F:GGACATCCTGGTGGATCTG*<br>*R:CCTGCTTGTATCTGTTGGTGAG*
NPC2	*F:TGGCAAATCCCAGTACAGATT*<br>*R:GCGTTCAGAGGCATGATACA*
PLA2G7	*F:GACATGGATCGTGGGAAGTC*<br>*R:GCAATACCGCACCTGAATCT*
BTG2	*F:GAGCAGCGACTCAAGGTTTT*<br>*R:CGATGCGATAGGACACTTCA*
RETSAT	*F:ACAAGGCGATGGAGCACTAC*<br>*R:TTCAACGAAGGAGCTTTTGAG*
FBP2	*F:TGGAGGGATCTTCCTGTACC*<br>*R:CTCGCTGATGAATGGTCTCA*
MYH2	*F:TCACCAAAGGCCAGACAGTA*<br>*R:TCCAAGACCCCGATGAAGTA*
CSRP3	*F:GTGCCATCTGTGGGAAGAGT*<br>*R:TGCTTTAGGCCAATGAGAGA*
PITX2	*F:TGGAGGCCACTTTCCAGAG*<br>*R:GTCGTCGTAGGGCTGCATAA*
TPM1	*F:CGACTGGCAACAGCTTTACA*<br>*R:TACGGGCCACCTCTTCATAC*
TNNI1	*F:CATGCCGGAAGTTGAGAGA*<br>*R:TCCACCTTGGCGTGAAGT*
MYOG	*F:GCTTTGAGCCACCAGGCTAC*<br>*R:CACAGACACGGACTTCCTCTT*
MYL3	*F:TGATGCCTCCAAGATCAAGA*<br>*R:GGCAGGAACGTGTCAAAGTC*
LMCD1	*F:CTCCGGCTGTGACGAGATA*<br>*R:CAGGATGGCCTTAGCACACT*
TMSB10	*F:TTCGATAAGGCCAAGCTGAA*<br>*R:CTTCCTCCACATCCCGATTA*
FHL1	*F:CCGAGTGCTTTGTGTGTGTT*<br>*R: GCCTTTACCAAACCCAGTGA*
TNNT1	*F:GACAGGGCGTGAGATGAAAC*<br>*R:CGCCATCAGGTCAAACTTCT*
LDHA	*F:AACACTGGAAAGCGGTTCAC*<br>*R: CCCAAGATGCAAGGAACACT*
ALDH2	*F:GCTGACCGTGGCTACTTCAT*<br>*R:CAGCCCGTACTTGGAATTGT*
GAPDH[a]	*F:TCACCAGGGCTGCTTTTAAC*<br>*R:ATGGCCTTTCCATTGATGAC*

[a] GAPDH is the internal control gene.
